# Association of Inflammatory Mediators with Mitochondrial DNA Variants in Geriatric COVID-19 Patients

**DOI:** 10.14336/AD.2023.1123

**Published:** 2024-02-09

**Authors:** Tiziana Casoli, Anna Rita Bonfigli, Mirko Di Rosa, Belinda Giorgetti, Marta Balietti, Robertina Giacconi, Maurizio Cardelli, Francesco Piacenza, Francesca Marchegiani, Fiorella Marcheselli, Rina Recchioni, Roberta Galeazzi, Salvatore Vaiasicca, Adrianapia Maria Lamedica, Alessia Fumagalli, Letizia Ferrara, Fabrizia Lattanzio

**Affiliations:** ^1^Center for Neurobiology of Aging, IRCCS INRCA, Ancona, Italy.; ^2^Scientific Direction, IRCCS INRCA, Ancona, Italy.; ^3^Centre for Biostatistics and Applied Geriatric Clinical Epidemiology, IRCCS INRCA, Ancona, Italy.; ^4^Advanced Technology Center for Aging Research, IRCCS INRCA, Ancona, Italy.; ^5^Clinic of Laboratory and Precision Medicine, IRCCS INRCA, Ancona, Italy.; ^6^Pulmonary Rehabilitation Unit, IRCCS INRCA, Casatenovo, Italy.; ^7^Medical Direction, IRCCS INRCA, Ancona, Italy.

**Keywords:** COVID-19, mtDNA, resequencing, homoplasmy, heteroplasmy, geriatric patients

## Abstract

COVID-19 remains a serious concern for elderly individuals with underlying comorbidities. SARS-CoV-2 can target and damage mitochondria, potentially leading to mutations in mitochondrial DNA (mtDNA). This study aimed to evaluate single nucleotide substitutions in mtDNA and analyze their correlation with inflammatory biomarkers in elderly COVID-19 patients. A total of 30 COVID-19 patients and 33 older adult controls without COVID-19 (aged over 65 years) were enrolled. mtDNA was extracted from buffy coat samples and sequenced using a chip-based resequencing system (MitoChip v2.0) which detects both homoplasmic and heteroplasmic mtDNA variants (40-60% heteroplasmy) and allows the assessment of low-level heteroplasmy (<10% heteroplasmy). Serum concentrations of IL-6, IFN-α, TNF-α and IL-10 were determined in patients by a high-sensitivity immunoassay. We found a higher burden of total heteroplasmic variants in COVID-19 patients compared to controls with a selective increment in *ND1* and *COIII* genes. Low-level heteroplasmy was significantly elevated in COVID-19 patients, especially in genes of the respiratory complex I. Both heteroplasmic variant burden and low-level heteroplasmy were associated with increased levels of IL-6, TNF-α, and IFN-α. These findings suggest that SARS-CoV-2 may induce mtDNA mutations that are related to the degree of inflammation.

Although the infection by SARS-CoV-2 is no longer a threatening issue and COVID-19 is not lethal as in the past two years, there are still concerns regarding the disease especially in older adults. Indeed, COVID-19 might have important complications leading to death for the over 80s, particularly those with comorbidities, namely coronary artery disease, chronic obstructive pulmonary disease, hypertension, obesity, and diabetes [1].

Recent evidence has established a direct connection between the outcomes of SARS-CoV-2 infection and the metabolic status of the host cells, with an intrinsic relationship between SARS-CoV-2 viral cycle and mitochondria [2, 3]. Upon infection, the organelles show changes in shape, structure, inner cristae-matrix interaction, and membrane potential [4]. Viral proteins and RNA localize to host cell mitochondria, a phenomenon referred to as viral hijacking of mitochondria [5, 6]. These events can lead to a major damage to mitochondria causing the release of the organelle components in the cytosol or in the extracellular compartment where they act as alarmins against cellular injury, to activate immune or inflammatory responses [7, 8]. These endogenous modulator molecules originating from mitochondria are collectively known as mitochondrial damage-associated molecular patterns (mtDAMP) and one of the most active in triggering inflammatory pathways is mitochondrial DNA (mtDNA) [9].

In elderly individuals, mitochondria exhibit notable functional decline characterized by reduced oxidative phosphorylation followed by decrease of ATP production along with a significant increase in the generation of reactive oxygen species (ROS). Additionally, there is an accumulation of mtDNA variants and a decrease in antioxidant defenses [10-12].

**Table 1:** Demographics, comorbidities, and blood test parameters at admission of controls and COVID-19 patients.

	Controls (n=33)	COVID-19 (n=30)	Age-weighted *p*-values
Demographic characteristics			
Age (years)	76.09 ± 6.45	87.47 ± 3.00	
Sex F, n (%)	23 (69.7%)	14 (46.7%)	0.064
Marital status, n (%)			0.221
*Single*	4 (12.1%)	1 (3.3%)	
*Widowed*	15 (45.6%)	8 (26.7%)	
*Married*	14 (42.4%)	21 (70.0%)	
Impaired ADL, median [IR]	0 (0-5)	5 (2-6)	0.804
Impaired IADL, median [IR]	1 (0-5)	5 (3-8)	0.742
Comorbidities			
Diabetes, n (%)	7 (21.2%)	5 (16.7%)	0.296
Stroke, n (%)	4 (12.1%)	2 (6.7%)	1.000
Cancer, n (%)	4 (12.1%)	7 (23.3%)	1.000
COPD, n (%)	4 (12.1%)	5 (16.7%)	0.296
Myocardial infarction, n (%)	4 (12.1%)	2 (6.7%)	0.296
Atrial fibrillation, n (%)	4 (12.1%)	10 (33.3%)	0.296
Alzheimer’s disease, n (%)	2 (6.1%)	8 (26.7%)	0.121
CKD, n (%)	11 (33.3%)	7 (23.3%)	0.221
Laboratory parameters			
D-Dimer (ng/ml)	1350.8 ± 890.5	2289.3 ± 3556.5	0.134
CRP (mg/l)	3.12 ± 2.69	3.99 ± 4.93	0.271
Hgb (g/dl)	11.21 ± 1.93	11.46 ±2.21	0.574
WBC*10^3^/µl	7.91 ± 2.90	9.56 ± 5.16	0.522
Neutrophils*10^3^/µl	5.78 ± 3.30	7.48 ± 3.83	0.150
Lymphocytes*10^3^/µl	1.62 ± 0.74	1.15 ± 0.56	0.078
Eosinophils*10^3^/µl	0.23 ± 0.21	0.13 ± 0.10	0.109
Basophils*10^3^/µl	0.03 ± 0.02	0.01 ± 0.01	0.236
Platelets*10^3^/µl	255.6 ± 147.7	245.3 ± 118.9	0.078
Ferritin (µg/l)	258.1 ± 327.1	667.1 ± 653.5	0.033
Creatinine (mg/dl)	1.56 ± 1.55	1.16 ± 0.68	0.809
AST (U/l)	22.08 ± 16.81	35.12 ± 25.47	0.100
ALT (U/l)	17.54 ± 8.79	30.84 ± 24.19	0.852
LDH (U/l)	186.0 ± 98.5	293.7 ± 108.2	0.562

ADL, activities of daily living; IADL, instrumental activities of daily living COPD, chronic obstructive pulmonary disease; CKD, Chronic Kidney Disease; CRP, C-reactive protein; Hgb, hemoglobin; WBC, white blood cells; AST, aspartate aminotrasferase; ALT, alanine aminotransferase; LDH, lactate dehydrogenase. Data are expressed as mean ± SD, or n (%). Unpaired Student’s t test and χ^2^ test were used to calculate statistical significance between groups. Age-weighted *p* values were determined by a logistic regression model. Values in bold are statistically significant *(p* ≤ 0.05).

The severity of COVID-19 in the elderly population is influenced by immunosenescence [13], an age-related dysfunction of the immune system that impairs its ability to fight infections and respond effectively to new antigens [14-16]. Furthermore, aging is associated with a proinflammatory state known as “inflammaging” [17], characterized by augmented activity of NF-κB, COX-2, and iNOS in activated cells like macrophages and dendritic cells. This leads to the release of proinflammatory cytokines such as interleukin 6 (IL-6), IL-1β, and tumor necrosis factor alpha (TFN-α) [18, 19], even in the absence of active diseases or overt infections. These changes contribute to a diminished efficacy of both the innate and adaptive immune responses and can result in further damage to the mitochondria during viral infections [11, 20].

Peripheral blood cells from patients with COVID-19 showed changes indicative of oxidative stress, such as increase of mitochondrial ROS (mtROS), decrease of superoxide dismutase activity and glutathione, and of α-ketoglutarate dehydrogenase levels [21, 22]. mtROS directly stimulate the production of proinflammatory cytokines [23] which in turn drive oxidative stress and ROS generation, leading to a vicious inflammatory and oxidation cycle in which damaged mitochondria cause further mitochondrial injury [24]. mtROS are the most recognized source of damage to mtDNA, characterized by single- and double-strand breaks, abasic sites, and oxidized bases, which are all believed to be important causes of mtDNA mutations [25, 26]. Therefore, it is plausible that the interaction between the SARS-CoV-2 and mitochondria could lead to specific mtDNA variants, especially in older patients. To verify this hypothesis, we conducted a study to examine the presence of homoplasmic and heteroplasmic single nucleotide substitutions in mtDNA of elderly individuals with COVID-19 and compared them to a control group. We also analyzed the potential association between the mtDNA variants and the inflammatory biomarkers in COVID-19 patients. The close relationship between SARS-CoV-2 infection and mitochondria could determine specific alterations of mtDNA which may persist even after COVID-19 recovery since the low efficiency of mitochondrial DNA repair system could hinder the restoration of mtDNA integrity.

**Table 2:** Clinical characteristics of COVID-19 patients.

COVID-19 symptoms and course	COVID-19 (n=30)
Days of clinic stay, median (IQR)	14 (8-23)
Respiratory insufficiency, n (%)	19 (63.3%)
Fever, median (IQR)	36.4 (36.0-36.6)
Cough, n (%)	10 (33.3%)
Diarrhea, n (%)	3 (10.0%)
Vomiting, n (%)	2 (6.7%)
Other infections, n (%)	0 (0.0%)
O_2_ saturation, median (IQR)	97 (95-98)
Anosmia, n (%)	1 (3.3%)
Dysgeusia, n (%)	2 (6.7%)
Deceased, n (%)	12 (40.0%)
Glucocorticoids, n (%)	2 (6.7%)
NSAID, n (%)	0 (0.0%)
Heparin, n (%)	2 (6.7%)

NSAID, non-steroidal anti-inflammatory drugs

## MATERIALS AND METHODS

### Study population

The study population comprised elderly COVID-19 inpatients (n=30, experimental group) and elderly hospitalized patients without COVID-19 as controls (n=33). Blood samples were collected from individuals who were admitted to the IRCCS INRCA Hospital, Ancona, Italy, and were enrolled in either the Report-Age COVID (experimental patients) or Report-Age (control subjects) project, respectively.

The aim of the Report-Age COVID project is to provide a deeper understanding of COVID-19 disease in elderly patients (≥ 65 years). All the included subjects were COVID-19 positive cases as confirmed by the detection of SARS-CoV-2 RNA in nasal/oropharyngeal swabs by reverse transcription-polymerase chain reaction. COVID-19 patients included in this study were admitted to IRCCS INRCA Hospital from 16th October 2020 to 31st December 2020. Demographic and anamnestic data, biochemical and hematological variables, information on treatments, comorbidities, and survival were collected in a retrospective manner and anonymized before release, as previously described [27]. The study protocol was approved by the local Ethics Committee (reference number CEINRCA-20008) and registered under the ClinicalTrials.gov database (reference number NCT04348396). The Report-Age project is a large-scale ongoing observational study focusing on the health conditions of hospitalized older adults at IRCCS INRCA Hospital and includes a high-level data resource of demographics, geriatric assessments, clinical and diagnostic information, comorbid health conditions, and frailty parameters, as well as biological samples [28]. Subjects enrolled in the present study were randomly selected. Control subjects with diagnosis of autoimmune or infectious diseases, from the Report-Age project, were excluded. The study protocol was approved by the local Ethics Committee (12/DSAN; 19 April 2011) and registered with Trial Registration no. NCT01397682. Both experimental patients and control subjects were not vaccinated.

**Table 3:** Haplogroup distribution expressed as absolute number and percentage.

	Total (n=63)	Controls (n=33)	COVID-19 (n=30)
H	23 (36.5%)	13 (39.4%)	10 (33.3%)
HV	3 (4.8%)	2 (6.1%)	1 (3.3%)
I	1 (1.6%)	0 (0%)	1 (3.3%)
J	5 (7.9%)	3 (9.1%)	2 (6.7%)
K	5 (7.9%)	2 (6.1%)	3 (10.0%)
N	1 (1.6%)	1 (3.0%)	0 (0.0%)
R	1 (1.6%)	1 (3.0%)	0 (0.0%)
T	10 (15.9%)	2 (6.1%)	8 (26.7%)
U	13 (20.6%)	8 (24.2%)	5 (16.7%)
V	1 (1.6%)	1 (3.0%)	0 (0.0%)

### mtDNA resequencing

DNA was extracted from buffy coat samples collected within 12 hours after patient admission. Briefly, EDTA blood samples were centrifuged at 2,500 x g at 4°C for 15 minutes, the buffy coat layer was removed and stored at -80°C. Total DNA was extracted by QIAamp DNA Blood Mini kit (Cat# 51104, Qiagen, Germany) according to the manufacturer’s instructions and kept at -20°C until use. mtDNA was obtained from the total DNA samples by specific amplification with the REPLI-g mitochondrial DNA kit (Cat# 151023, Qiagen, Germany). After purification, DNA was quantified spectrophotometrically and the Genechip Resequencing array kit (Cat# 900447, Thermofisher Scientific, USA) was used for mtDNA fragmentation and staining. Briefly, the labeled mtDNA was loaded onto MitoChip v2.0 arrays (Cat# 900886, Thermo Fisher Scientific, USA), which underwent washing and staining in the Fluidics Station 450, followed by scanning using the Affymetrix GeneChip Scanner 3000 7G.

The data analysis was performed using the GeneChip Sequence Analysis Software (GSEQ) 4.1. For this study, two types of files were used: “Single Nucleotide Polymorphism (SNP) View”, which compared the obtained data with the revised Cambridge Reference Sequence (rCRS) to identify homoplasmic and heteroplasmic variants, and “Probe Intensity”, which provided fluorescence intensity values of the four bases for each nucleotide position (np) in both the sense and antisense strands. Heteroplasmic variants were identified by GSEQ 4.1 based on a range of 40-60% heteroplasmy. The number of homoplasmic and heteroplasmic variants was normalized by gene length and expressed as variant burden.

The Probe Intensity files were used to calculate the Ratio of Expected Allele (REA), which represents the log ratio of the fluorescence intensity values of the reference nucleotide, as indicated in the rCRS, to the average signal intensity of the other three nucleotides. This measurement quantifies allelic substitutions in each np and is used for the assessment of low-level (< 10%) heteroplasmy [29]. The complete microarray data for this study can be accessed at Gene Expression Omnibus (GEO accession number: GSE235626). Free bioinformatic tools were used to identify missense variants (http://mitotool.kiz.ac.cn/) and mtDNA haplogroups (https://dna.jameslick.com/).

### In silico prediction of mutation pathogenicity

Five online tools were used to evaluate the possible pathogenicity of missense variants in affected genes: PolyPhen 2 HumVar (http://genetics.bwh.harvard.edu/ pph2/), PANTHER (www.pantherdb.org/tools/), SIFT (http://sift-dna.org), MutPred2 (http://mutpred.mutdb.org/), and Align-GVGD (http://agvgd.hci.utah.edu/). PolyPhen 2 HumVar is a tool that predicts the possible impact of amino acid substitutions on protein structure and function by using physical and comparative analyses. Features such as atomic contacts and solvent accessibility are assessed, and empirically determined cut-offs are used to predict pathogenicity. PANTHER employs an evolutionary analysis that estimates the likelihood that a missense SNP determines a functional impact on the protein. Homologous proteins are used to reconstruct the likely sequences of ancestral proteins, and the history of each amino acid can be traced back in time to estimate how long that state has been preserved, the longer the time, the more damaging the substitution. SIFT is an algorithm which predicts whether an amino acid substitution affects protein function based on sequence homology and physical properties of amino acids. MutPred2 is a machine learning-based method that integrates genetic and molecular data to predict probabilistically the pathogenicity of amino acid substitutions. This is achieved by providing a ranked list of specific molecular alterations potentially influencing the phenotype including secondary structure, signal peptide and transmembrane topology, catalytic activity, and allostery. Align-GVGD combines the biophysical characteristics of amino acids and multiple protein sequence alignments to predict the effect of missense substitutions. The algorithm calculates two variables that, together, are related to substitution severity, GV and GD. These are used in a formula that allows the definition of the predicted effect as C0, C15, C25, C35, C45, C55, or C65, with C65 meaning “most likely to interfere with function” and C0 “least likely pathogenetic”.

**Figure 1.**
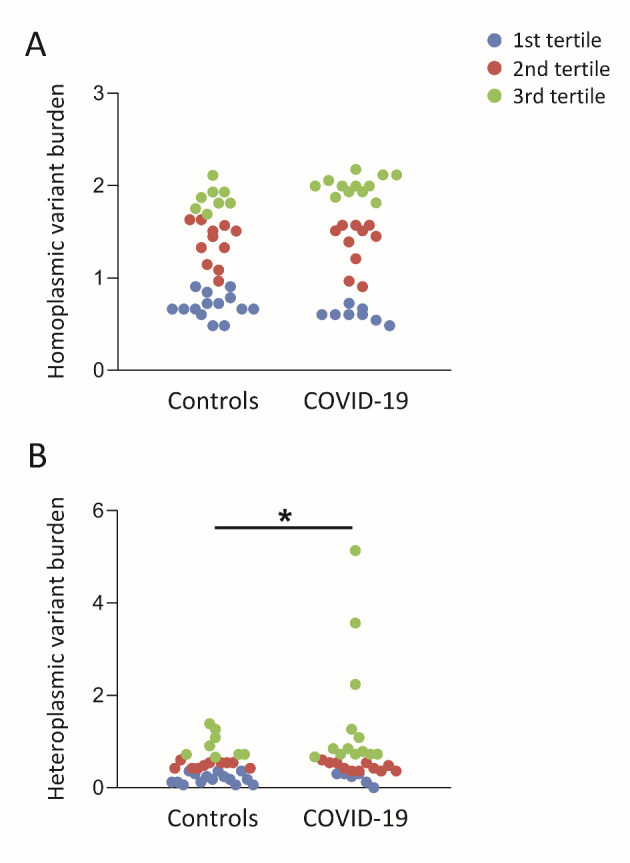
**Homoplasmic and heteroplasmic mtDNA variant burden in controls and COVID-19 patients**. Subjects (controls, n=33; COVID-19, n=30) were divided in tertiles according to variant burden amount (1st tertile, low; 2nd tertile, medium; 3rd tertile, high). **(A)** Distribution of homoplasmic variant burden in control and COVID-19 subjects. Comparison between the two groups did not result statistically significant. **(B)** The heteroplasmic variant burden in the COVID-19 group was significantly different from controls showing a decrease of the 1st and an increase of the 3rd tertile. Group differences were analyzed by age-adjusted Pearson’s χ^2^ test (* *p* ≤ 0.05).

**Table 4:** Tertile distribution of homoplasmic variant burden per gene.

	Controls (n=33)	COVID-19 (n=30)	Age-weighted *p*-values
homo_*D-loop*			0.002
1	10 (30.3%)	12 (40.0%)	
2	6 (18.2%)	14 (46.7%)	
3	17 (51.5%)	4 (13.3%)	
homo_*12S*			1.000
1	28 (84.8%)	18 (60.0%)	
2	0 (0.0%)	0 (0.0%)	
3	5 (15.2%)	12 (40.0%)	
homo_*16S*			0.393
1	9 (27.3%)	12 (40.0%)	
2	10 (30.3%)	14 (46.7%)	
3	14 (42.4%)	4 (13.3%)	
homo_*ND1*			0.549
1	17 (51.5%)	9 (30.0%)	
2	14 (42.4%)	17 (56.7%)	
3	2 (6.1%)	4 (13.3%)	
homo_*ND2*			0.766
1	14 (42.4%)	11 (36.7%)	
2	14 (42.4%)	12 (40.0%)	
3	5 (15.2%)	7 (23.3%)	
homo_*COI*			0.558
1	26 (78.8%)	20 (66.7%)	
2	0 (0.0%)	0 (0.0%)	
3	7 (21.2%)	10 (33.3%)	
homo_*COII*			1.000
1	29 (87.9%)	26 (86.7%)	
2	0 (0.0%)	0 (0.0%)	
3	4 (12.1%)	4 (13.3%)	
homo_*ATPase8*			1.000
1	32 (97.0%)	28 (93.3%)	
2	0 (0.0%)	0 (0.0%)	
3	1 (3.0%)	2 (6.7%)	
homo_*ATPase6*			0.400
1	22 (66.7%)	17 (56.7%)	
2	11 (33.3%)	10 (33.3%)	
3	0 (0.0%)	3 (10.0%)	
homo_*COIII*			0.393
1	24 (72.7%)	16 (53.3%)	
2	7 (21.2%)	13 (43.3%)	
3	2 (6.1%)	1 (3.3%)	
homo_*ND3*			0.296
1	29 (87.9%)	25 (83.3%)	
2	0 (0.0%)	0 (0.0%)	
3	4 (12.1%)	5 (16.7%)	
homo_*ND4L*			0.296
1	31 (93.9%)	28 (93.3%)	
2	0 (0.0%)	0 (0.0%)	
3	2 (6.1%)	2 (6.7%)	
homo_*ND4*			0.209
1	14 (42.4%)	9 (30.0%)	
2	11 (33.3%)	11 (36.7%)	
3	8 (24.2%)	10 (33.3%)	
homo_*ND5*			0.264
1	12 (36.4%)	9 (30.0%)	
2	16 (48.5%)	15 (50.0%)	
3	5 (15.2%)	6 (20.0%)	
homo_*ND6*			1.000
1	24 (72.7%)	20 (66.7%)	
2	0 (0.0%)	0 (0.0%)	
3	9 (27.3%)	10 (33.3%)	
homo_*Cytb*			0.788
1	19 (57.6%)	15 (50.0%)	
2	9 (27.3%)	7 (23.3%)	
3	5 (15.2%)	8 (26.7%)	

### Measurement of inflammatory biomarkers

Upon collection, serum tubes of COVID-19 patients were gently inverted eight times and left at room temperature for 60 minutes to allow clotting. Subsequently, the tubes were centrifuged at 2500 x g at 4°C for 15 minutes. After centrifugation, the top portion of the supernatant was carefully aspirated and stored at -80°C in aliquots of 0.5-1 ml. The serum concentration of IL-6, IFN-α, TNF-α, and IL-10 was determined using Pro Quantum Immunoassays (Cat# A35573, A42897, A35601, A35590, Thermo Fisher Scientific, USA), with each molecule having a specific assay. Duplicate reactions were performed for each sample, using 2 μl of serum, and the assays were run on Aria Mx Real Time PCR Instrument (Agilent, USA), following the conditions specified by the manufacturer.

### Statistical analysis

All the tests, comparing COVID-19 patients and controls, were conducted addressing the potential bias arising from the different mean age of the two groups by the application of a propensity score matching (PSM) method. Age-weighted matching was performed by estimating the propensity scores through a stepwise logistic regression model, with the patient's group (COVID-19 or control) as the dependent variable and age as a covariate.

**Table 5:** Tertile distribution of heteroplasmic variant burden per gene.

	Controls (n=33)	COVID-19 (n=30)	Age-weighted *p*-values
het_ *D-loop*			0.513
1	15 (45.5%)	16 (53.3%)	
2	7 (21.2%)	6 (20.0%)	
3	11 (33.3%)	8 (26.7%)	
het_*12S*			0.221
1	26 (78.8%)	19 (63.3%)	
2	0 (0.0%)	0 (0.0%)	
3	7 (21.2%)	11 (36.7%)	
het_*16S*			0.505
1	24 (72.7%)	23 (76.7%)	
2	0 (0.0%)	0 (0.0%)	
3	9 (27.3%)	7 (23.3%)	
het_*ND1*			0.003
1	28 (84.8%)	19 (63.3%)	
2	0 (0.0%)	0 (0.0%)	
3	5 (15.2%)	11 (36.7%)	
het_*ND2*			0.135
1	23 (69.7%)	17 (56.7%)	
2	5 (15.2%)	6 (20.0%)	
3	5 (15.2%)	7 (23.3%)	
het_*COI*			0.221
1	24 (72.7%)	18 (60.0%)	
2	0 (0.0%)	0 (0.0%)	
3	9 (27.3%)	12 (40.0%)	
het_*COII*			0.296
1	28 (84.8%)	20 (66.7%)	
2	0 (0.0%)	0 (0.0%)	
3	5 (15.2%)	10 (33.3%)	
het_*ATPase8*			0.296
1	32 (97.0%)	29 (96.7%)	
2	0 (0.0%)	0 (0.0%)	
3	1 (3.0%)	1 (3.3%)	
het_*ATPase6*			0.296
1	30 (90.9%)	24 (80.0%)	
2	0 (0.0%)	0 (0.0%)	
3	3 (9.1%)	6 (20.0%)	
het_*COIII*			0.050
1	24 (72.7%)	5 (16.7%)	
2	6 (18.2%)	10 (33.3%)	
3	3 (9.1%)	15 (50.0%)	
het_*ND3*			0.121
1	33 (100.0%)	24 (80.0%)	
2	0 (0.0%)	0 (0.0%)	
3	0 (0.0%)	6 (20.0%)	
het_*ND4L*			0.121
1	32 (97.0%)	26 (86.7%)	
2	0 (0.0%)	0 (0.0%)	
3	1 (3.0%)	4 (13.3%)	
het_*ND4*			0.264
1	19 (57.6%)	11 (36.7%)	
2	9 (27.3%)	13 (43.3%)	
3	5 (15.2%)	6 (20.0%)	
het_*ND5*			0.076
1	21 (63.6%)	19 (63.3%)	
2	7 (21.2%)	5 (16.7%)	
3	5 (15.2%)	6 (20.0%)	
het_*ND6*			0.221
1	29 (87.9%)	22 (73.3%)	
2	0 (0.0%)	0 (0.0%)	
3	4 (12.1%)	8 (26.7%)	
het_*Cytb*			0.558
1	24 (72.7%)	20 (66.7%)	
2	0 (0.0%)	0 (0.0%)	
3	9 (27.3%)	10 (33.3%)	

Data are expressed as patient number in the single tertile (%). Data were analyzed by age-adjusted Pearson’s χ^2^ test. Values in bold are statistically significant (*p* ≤ 0.05).

Participant characteristics were reported as mean and standard deviation for continuous variables, after their normality had been assessed using the Shapiro-Wilk test, and as absolute frequency and percentage for categorical variables. The comparison of variables between groups was performed using unpaired Student’s t test and χ^2^ test for continuous and categorical variables, respectively.

Tertiles of homoplasmic and heteroplasmic variant burden, both total and per single gene, were calculated and expressed as absolute number and percentages. The tertiles were arranged in ascending order, with the first tertile representing subjects with a lower value of variant burden, and were compared by Pearson’s χ^2^ test.

REA values were calculated for each of the 16,544 np in every subject and averaged for each np in the two groups. The comparison of REA values between the groups was performed using an unpaired Student's t test or Mann-Whitney U test, depending on the data distribution. For both tests, np with a *p* ≤ 0.05 and a mean absolute difference ≥ 0.25 were selected. To account for multiple testing and control for false significant comparisons, the Benjamini-Hochberg correction was applied.

To explore associations between mtDNA variants in COVID-19 patients and inflammatory biomarkers (IL-6, IFN-α, TNF-α, and IL-10), Spearman rank correlation with Bonferroni correction was employed. All data analyses were conducted using STATA version 15.1 Statistical Software Package for Windows (StataCorp, College Station, TX). Significance level was set at *p* ≤ 0.05.

## RESULTS

### Study group characteristics

Demographics, comorbidities, and laboratory parameters of the study population are shown in Table 1. The age of COVID-19 and control groups differed significantly; therefore, all the comparisons were conducted applying age-adjustment. Both groups were thoroughly examined for comorbidities including diabetes, stroke, cancer, chronic obstructive pulmonary disease (COPD), myocardial infarction, atrial fibrillation, Alzheimer’s disease, and chronic kidney disease (CKD). No significant differences were observed. Ferritin levels resulted significantly higher in COVID-19 patients, while all the other laboratory parameters were not statistically different between the two groups. Table 2 summarizes clinical characteristics of COVID-19 patients. They had a median hospitalization duration of 14 days and 40% of them deceased. The most common symptoms were respiratory insufficiency (63.3%) and cough (33.3%), followed by gastrointestinal symptoms (diarrhea: 10.0%; vomiting: 6.7%), and dysgeusia (6.7%). Treatments at admission included glucocorticoids and heparin. The clinical features of our cohort were in line with those reported in unvaccinated patients of the same age group [30, 31]. The distribution of mtDNA haplogroups is shown in Table 3. No difference was found between the two groups investigated (*p* = 0.504).

**Figure 2.**
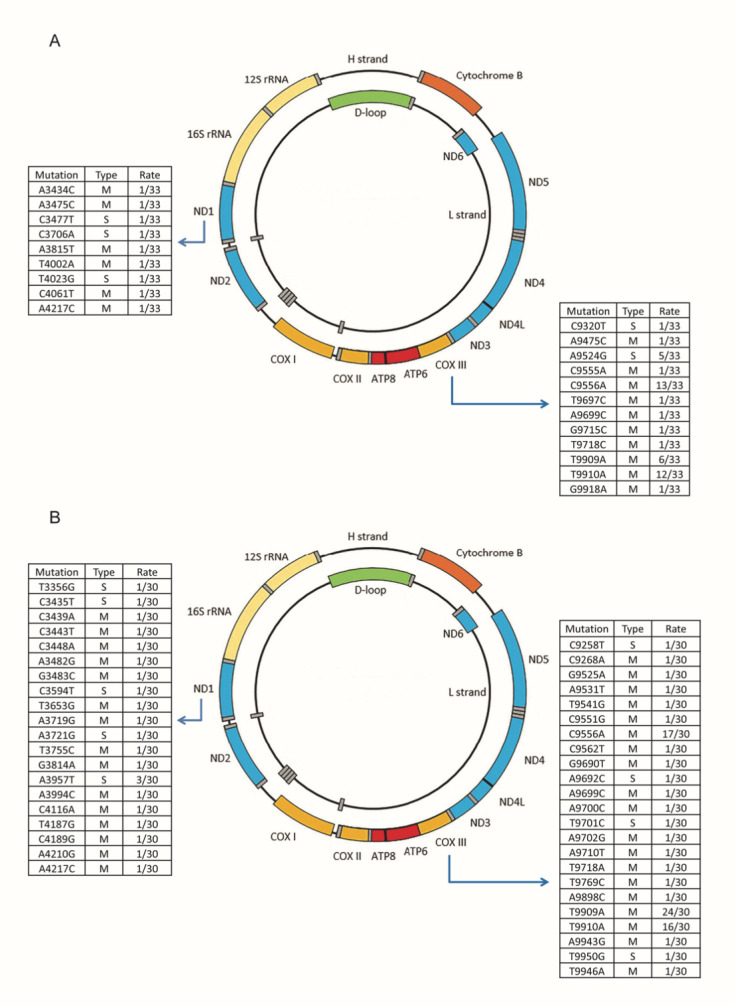
**Type and rate of heteroplasmic variants in *ND1* and *COIII* genes. (A)** Heteroplasmic variants in the control group (n=33). mtDNA is represented as two circles, the H strand and the L strand, reporting in color the coding genes and the D-loop region. Single nucleotide substitutions in *ND1* and *COIII* genes, as well as type (missense or silent variant) and rate in the studied group are shown in the corresponding tables. **(B)** Heteroplasmic variants in the COVID-19 group (n=30) are reported. It can be observed the increase of variant number in comparison to control group. M, missense variants; S, silent variants.

**Table 6:** Missense *ND1* gene mutations in controls and COVID-19 patients, amino acid substitutions, and predicted pathogenicity scores.

Mutation	Amino acid	PolyPhen-2 HumVar	PANTHER	SIFT	MutPred2	Align-GVGD
Controls						
A3434C	Y43S	Possibly damaging	Probably damaging	Deleterious	Pathogenic	C65
A3475C	T57P	Probably damaging	Probably damaging	Deleterious	Benign	C35
**A3815T**	**E170V**	**Probably damaging**	**Probably damaging**	**Deleterious**	**Pathogenic**	**C65**
T4002A	I232M	Possibly damaging	Probably damaging	Tolerated	Pathogenic	C0
C4061T	P252L	Possibly damaging	Probably damaging	Tolerated	Benign	C65
A4217C	Y304S	Benign	Probably damaging	Tolerated	Benign	C65
COVID-19						
C3439A	L45M	Possibly damaging	Probably damaging	Deleterious	Benign	C0
**C3443T**	**L46P**	**Probably damaging**	**Probably damaging**	**Deleterious**	**Pathogenic**	**C65**
C3448A	P48T	Probably damaging	Probably damaging	Deleterious	Benign	C35
A3482G	E59G	Probably damaging	Probably damaging	Deleterious	Benign	C65
G3483C	E59D	Probably damaging	Probably damaging	Deleterious	Benign	C35
**T3653G**	**I116S**	**Probably damaging**	**Probably damaging**	**Deleterious**	**Pathogenic**	**C65**
A3719G	Q138R	Probably damaging	Probably damaging	Deleterious	Pathogenic	C35
**T3755C**	**L150P**	**Probably damaging**	**Probably damaging**	**Deleterious**	**Pathogenic**	**C65**
G3814A	E170K	Probably damaging	Probably damaging	Tolerated	Benign	C55
**A3994C**	**N230H**	**Probably damaging**	**Probably damaging**	**Deleterious**	**Pathogenic**	**C65**
C4116A	F270L	Possibly damaging	Probably damaging	Deleterious	Benign	C15
**T4187G**	**L294R**	**Probably damaging**	**Probably damaging**	**Deleterious**	**Pathogenic**	**C65**
C4189G	P295A	Probably damaging	Probably damaging	Deleterious	Benign	C25
A4210G	M302V	Probably damaging	Probably damaging	Tolerated	Benign	C15
A4217C	Y304S	Benign	Probably damaging	Tolerated	Benign	C65

In bold missense mutations with the highest scores of pathogenicity in all the five tools used. Align-GVGD score should be interpreted as C0 = less likely pathogenic; C65 = most likely pathogenic.

### COVID-19 patients show a selective increase of heteroplasmic variant burden in ND1 and COIII genes

The analysis of variant burden, including both homoplasmic and heteroplasmic ones, revealed a significantly higher burden of heteroplasmic variants in COVID-19 patients when compared to controls (Fig.1). Specifically, the percentage of patients in the third tertile showed a significant increase. We then examined the tertile distribution of variant burden per single gene. We did not include in our analysis tRNA genes and the short non-coding sequences. The comparisons of homoplasmic variant burden did not yield significant differences, except for the D-loop region where controls exhibited an increased variant burden (Table 4). As regards heteroplasmic variant burden, an increase was observed in COVID-19 patients for the NADH dehydrogenase subunit 1 (*ND1*) and cytochrome c oxidase subunit III (*COIII*) genes (Table 5). The *ND1* gene is part of complex I, while *COIII* belongs to complex IV of the mitochondrial respiratory chain. In order to delve deeper into these regions, we conducted a detailed analysis, examining the type and rate of variants, as well as the presence of any amino acid change in the corresponding proteins (Fig. 2).

While *ND1* gene showed isolated variants, *COIII* gene displayed repeated variants in specific np, both in controls and COVID-19 patients. Specifically, 94.8% and 86.3% of the variants in the *ND1* gene, and 72.7% and 77.7% of the variants in the *COIII* gene led to an amino acid change in COVID-19 patients and controls, respectively. However, these differences within genes were not statistically significant.

The actual number of mtDNA homoplasmic and heteroplasmic variants per individual and their distribution in different genes are reported in Supplementary Table 1.

### Predicted effect on protein function of missense mutations in ND1 and COIII genes

The outcomes of in silico analysis of *ND1* and *COIII* missense variants are reported in Table 6 and Table 7, respectively. Both genes showed different pathogenic scores according to the algorithm used, except for PANTHER reporting all missense mutations as “probably damaging”. Table 6 shows that in ND1 gene, one substitution in controls, and five in COVID-19 patients, are associated with the maximum score of pathogenicity in all the tools considered. Table 7 displays the results of the analysis for *COIII*: maximum scores are reported in two SNP in controls, and in four SNP in COVID-19 patients. One of them, the C9556A, was common to both groups.

**Table 7:** Missense *COIII* gene mutations in controls and COVID-19 patients, amino acid substitutions, and predicted pathogenicity scores.

Mutation	Amino acid	PolyPhen-2 HumVar	PANTHER	SIFT	MutPred2	Align- GVGD
Controls						
**A9475C**	**E90A**	**Probably damaging**	**Probably damaging**	**Deleterious**	**Pathogenic**	**C65**
C9555A	P117T	Probably damaging	Probably damaging	Deleterious	Pathogenic	C35
**C9556A**	**P117H**	**Probably damaging**	**Probably damaging**	**Deleterious**	**Pathogenic**	**C65**
T9697C	L164P	Possibly damaging	Probably damaging	Tolerated	Pathogenic	C65
A9699C	I165L	Benign	Probably damaging	Tolerated	Benign	C0
G9715C	G170A	Probably damaging	Probably damaging	Tolerated	Pathogenic	C55
T9718C	L171H	Possibly damaging	Probably damaging	Deleterious	Pathogenic	C65
T9909A	F235I	Probably damaging	Probably damaging	Deleterious	Pathogenic	C15
T9910A	F235Y	Possibly damaging	Probably damaging	Tolerated	Benign	C15
G9918A	A238T	Probably damaging	Probably damaging	Tolerated	Benign	C55
COVID-19						
C9268A	A21D	Probably damaging	Probably damaging	Deleterious	Benign	C65
G9525A	A107T	Probably damaging	Probably damaging	Tolerated	Benign	C55
A9531T	T109S	Probably damaging	Probably damaging	Tolerated	Benign	C55
T9541G	L112W	Probably damaging	Probably damaging	Deleterious	Pathogenic	C55
C9551G	H115Q	Probably damaging	Probably damaging	Tolerated	Benign	C15
**C9556A**	**P117H**	**Probably damaging**	**Probably damaging**	**Deleterious**	**Pathogenic**	**C65**
C9562T	T119M	Probably damaging	Probably damaging	Tolerated	Benign	C65
G9690T	A162S	Probably damaging	Probably damaging	Tolerated	Benign	C65
A9699C	I165L	Benign	Probably damaging	Tolerated	Benign	C0
A9700C	I165T	Benign	Probably damaging	Deleterious	Benign	C65
A9702G	T166A	Probably damaging	Probably damaging	Deleterious	Benign	C55
A9710T	L168F	Probably damaging	Probably damaging	Tolerated	Benign	C15
T9718A	L171H	Possibly damaging	Probably damaging	Deleterious	Pathogenic	C65
T9769C	I188T	Probably damaging	Probably damaging	Deleterious	Benign	C65
**A9898C**	**H231P**	**Probably damaging**	**Probably damaging**	**Deleterious**	**Pathogenic**	**C65**
T9909A	F235I	Probably damaging	Probably damaging	Deleterious	Pathogenic	C15
T9910A	F235Y	Possibly damaging	Probably damaging	Tolerated	Benign	C15
**A9943G**	**D246G**	**Probably damaging**	**Probably damaging**	**Deleterious**	**Pathogenic**	**C65**
**T9946A**	**V247E**	**Probably damaging**	**Probably damaging**	**Deleterious**	**Pathogenic**	**C65**

In bold missense mutations with the highest scores of pathogenicity in all the five tools used. Align-GVGD score should be interpreted as C0 = less likely pathogenic; C65 = most likely pathogenic.

### mtDNA low-level heteroplasmy is increased in COVID-19 patients

**Figure 3.**
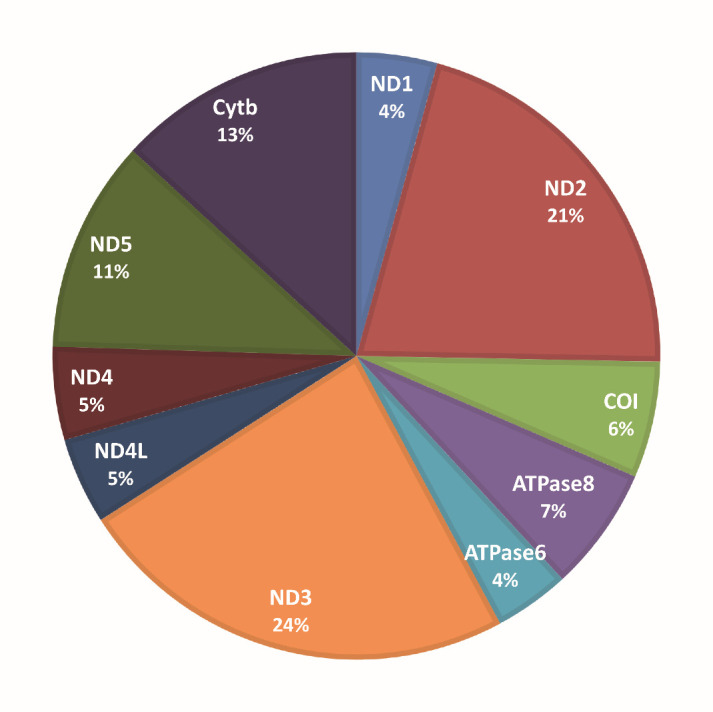
**Percentage distribution in coding genes of the 94 nucleotide positions showing increased low-level heteroplasmy in COVID-19 patients**. The pie graph shows that the most represented genes with increased low-level heteroplasmy in COVID-19 patients (n=30) vs. controls (n=33) are those of complex I of the respiratory chain (NADH-ubiquinone oxidoreductase) from *ND1* to *ND5*. The highest frequency was found in *ND2* and *ND3* genes.

Low-level heteroplasmy, defined as the presence of non-reference alleles in less than 10% of mtDNA copies, may be an indicator of non-specific acquired variants potentially induced by environmental and pathogenetic factors. To assess low-level heteroplasmy in mtDNA, we utilized the REA index at the single np level. High REA values indicate a prevalence of the reference nucleotide, whereas low values suggest a significant contribution of the other three nucleotides. The REA indices were determined to every np and compared between controls and COVID-19 patients. Out of the total 16,544 nucleotide positions analyzed, we found that 120 positions exhibited statistically significant differences in REA values between the control group and COVID-19 patients (Supplementary Table 2). REA was higher in controls in all of the 120 np, demonstrating, in the same np, a greater contribution of non-reference alleles in COVID-19 patients, i.e. an increase of low-level heteroplasmy. Among these 120 positions, 94 np belonged to coding genes; their relative frequency in the corresponding gene, after normalization by gene length, is illustrated in Fig. 3. Notably, the most represented genes are *ND2* and *ND3*, accounting respectively for 21% and 24% of significantly different np.

### Single nucleotide substitutions in mtDNA sequence associate with levels of inflammatory mediators in COVID-19 patients

Potential associations within COVID-19 group between heteroplasmic variant burden, along with the averaged REA values, and inflammatory mediators such as IL-6, IFN-α, TNF-α, and IL-10 were analyzed by Spearman rank correlation analysis. We investigated mtDNA genes resulting significantly different from controls. To account for multiple testing, we applied the Bonferroni correction. The results are summarized in Table 8. Analysis of the correlation coefficients (*r* scores) highlighted some significant correlations: positive between total and *ND1* gene heteroplasmic variant burden and IFN-α, and negative between REA values of many genes, and IL-6 and TNF-α. These findings indicate that an increase in heteroplasmic variant burden correlates specifically with an increase of IFN-α, while low REA scores, suggestive of low-level heteroplasmy, are associated with high levels of IL-6 and TNF-α.

## DISCUSSION

The main finding of this study is that elderly patients diagnosed with COVID-19 display higher heteroplasmic single nucleotide substitutions in their mtDNA compared to controls, both at high and low degree of heteroplasmy, and these mtDNA substitutions are associated with blood levels of inflammatory cytokines. The laboratory findings showed higher levels of ferritin in COVID-19 patients with respect to control group while other parameters investigated did not differ significantly. During viral infections, the concentration of circulating ferritin rises indicating viral replication and cytokine storm [32, 33]. Our results are in line with other laboratory test results showing a high variability within COVID-19 group and it has been demonstrated that they change according to disease severity and prognosis [27, 34, 35].

The analysis of mtDNA sequences was performed by using MitoChips which contains tens of thousands of oligonucleotide sequences, each representing a part of a mitochondrial gene, as spots on a solid surface. This technique is based on hybridization while next-generation sequencing (NGS) relies on synthesis by DNA polymerase to incorporate complementary nucleotides. MitoChip assay is well validated and has been demonstrated to provide reproducible results [36, 37]. Comparing MitoChip to capillary electrophoresis sequencing showed a concordance between both methods of 99.9% [38]. Another important advantage of using MitoChip is that the sequencing of nuclear mitochondrial DNA segments (NUMTs) is highly unlikely due to the amplification of sufficiently long mtDNA fragments that are not duplicated in the nuclear genome [29].

We found that total heteroplasmic variant burden was increased in patients compared to controls and the most important contribution to this increment was due to *ND1* and *COIII* genes. Surprisingly, the majority of observed variants were transversions. The empirically observed transition/transversion ratio is 21.97 in the mtDNA overall [39], but heteroplasmic changes show a higher proportion of transversion events [40]. In addition, the mtDNA analyzed in this study, deriving from middle-old and oldest-old individuals, might have been subjected to repeated events of oxidative stress, resulting in increase of transversions, although this issue has been strongly questioned in recent years, [41, 42]. Two previous investigations studied mtDNA variants associated with COVID-19 and they both demonstrated an increment in patient group vs. controls [43, 22]. Specifically, Dirican and colleagues [43] by Sanger sequencing of two specific genes, *ATPase6* and *Cytb*, found that COVID-19 patients displayed more A8860G and G9055A substitutions in the *ATPase6* gene and more A15326G, T15454C, and C15452A changes in the *Cytb* gene. Kumari et al., analyzed whole mtDNA genome by NGS and discovered 15 highly conserved and pathogenic variants associated with COVID-19 in genes of mitochondrial complex I and complex IV [22]. Interestingly, we also found an increase of heteroplasmic variant burden in COVID-19 patients in genes of the same respiratory complexes. The presence of a significant number of predicted pathogenic variants suggests that changes in amino acids may result in change of charge, hydrophobicity/hydrophilicity, and conformation of the secondary, tertiary, or quaternary structure, ultimately affecting the protein function [44]. As these complexes are engaged in different functions other than electron transport, such as mitochondrial morphology, calcium homeostasis, regulation of apoptosis, and ROS production, it can be inferred that mitochondrial activity could be deeply perturbated by their modification [45, 46].

The D-loop region, the non-coding region where mtDNA duplication starts, shows an increased homoplasmic variant burden in controls. Homoplasmic variants are mostly inherited and are particularly frequent in this region compared to ribosomal, tRNA, and coding mtDNA genes. They could be haplogroup markers, although no difference in haplogroup distribution was found between the two groups. It has been demonstrated that D-loop inherited variability plays a role in successful aging and longevity [47-49], however the role played by D-loop homoplasmic variants in COVID-19 affecting elderly individuals should be further evaluated. The increase of mtDNA low-level heteroplasmy in COVID-19 patients, demonstrated by the low REA values found in this group, support further the association of the disease with mtDNA modifications. The analysis of the relative frequency showed that *ND2* and *ND3* genes displayed lower-level heteroplasmy than other genes, indicating again the involvement of complex I in COVID-19 manifestations. An increase of low-level heteroplasmy has been demonstrated in aging and in age-associated neurodegenerative diseases like Alzheimer’s disease and Parkinson’s disease [50-52]. Currently, it is not solidly defined whether these variants are causal or correlative with aging, but strong indications suggest that they contribute to oxidative phosphorylation dysfunction and some aging phenotypes like the loss of muscle fibers and the replicative senescence [53].

**Table 8:** Correlation *r* values between heteroplasmic mutation burden and REA values of genes significantly different from controls, and IL-6, IFN-α, TNF-α, and IL-10 levels.

	IL-6	IFN-α	TNF-α	IL-10
het_mtDNA	0.3056	**0.4249**	-0.0341	-0.1417
het_*ND1*	0.0549	**0.4004**	0.0323	-0.1808
het_*COIII*	0.0458	0.3154	0.1100	-0.2879
REA_*ND1*	-0.3428	-0.0782	-0.2697	-0.1395
REA_*ND2*	-**0.3673**	-0.1626	-0.163	-0.0078
REA_*COI*	-0.3709	0.0435	-0.3025	-0.2187
REA_*ATPase8*	-**0.4154**	-0.2528	-0.0895	-0.0042
REA_*ATPase6*	-**0.4256**	-0.0580	-**0.3838**	-0.2058
REA_*ND3*	-0.3299	-0.1919	-0.1945	-0.0176
REA_*ND4L*	-**0.4772**	-0.2149	-0.2653	-0.1493
REA_*ND4*	-0.3477	-0.0449	-0.3363	-0.2040
REA_*ND5*	-**0.4532**	-0.0440	-0.2949	-0.1706
REA_*Cytb*	-0.3473	-0.0092	-0.2987	-0.1746

Data were analyzed by Spearman rank correlation with Bonferroni correction (n=30). Values in bold are statistically significant (*p* ≤ 0.05).

The association of IL-6, TNF-α, and IFN-α levels with mtDNA variants, at different degree of heteroplasmy, suggests a possible link between inflammation and mtDNA damage. Medications or concomitant infections could directly influence inflammatory mediator levels and their association with mtDNA variants. In this study, only 2/30 subjects took glucocorticoids or heparin, and none of them took NSAID. No one had coexisting infections. However, the influence of other possible factors on the association of inflammatory mediators with mtDNA variants should be evaluated. Thus, further large-scale studies are required to elucidate the role of inflammation on mtDNA variant increase in COVID-19, with better adjustment of confounding factors.

A highly proinflammatory microenvironment has been correlated with mitochondrial mutagenesis in synovial tissue of patients with arthritis [54]. Synovial tissue mtDNA mutation frequency significantly correlated with synovial fluid expression of TNF-α and IFN-γ demonstrating a strong relationship between mitochondrial instability and proinflammatory pathways. Another condition characterized by high levels of inflammation, caused by a dysregulated host response to infection, is sepsis. Park and colleagues demonstrated, by NGS of the complete mitochondrial genome, that PBMC from pediatric patients with severe sepsis or septic shock had a high incidence of mtDNA mutations [55]. In this regard, also COVID-19 is a condition characterized by high levels of pro-inflammatory cytokines and chemokines, which can promote not only local tissue inflammation but also a systemic inflammatory response, especially in older individuals [56]. The association of circulating cytokines with mtDNA single nucleotide substitutions suggests that there may exist a link between the inflammatory response elicited by Sars-CoV-2 infection and mitochondria. Indeed, interactions of the SARS-CoV-2 proteins such as open reading frames (ORF) and virus non-structural proteins (NSP) with host cell mitochondrial proteins lead to loss of membrane integrity and cause dysfunction in the bioenergetics of the mitochondria [3, 57]. What follows is a secondary damaging event with the release of mtDNA, elevated ROS, activation of the NF-κB pathway, inflammasomes, and hyper-inflammatory state and cytokine storm [58, 59]. Although the production of ROS in inflammatory conditions provides a host protective role, evidence suggests that their increase leads to the destruction of extracellular matrix components and oxidative damage of DNA molecules, especially the highly susceptible mtDNA [60]. Exposure to nitric oxide and peroxynitrite causes mtDNA damage and results in decreased cellular ATP levels and mitochondrial redox function [61]. In chronic liver inflammation, ROS overproduction directly caused mtDNA mutations [62]. Since expression of the entire mitochondrial genome is necessary for the maintenance of mitochondrial functions, including electron transport, small changes in mtDNA sequence can result in profound impairment of such functions, thereby enhancing generation of free radicals, which in turn accelerates the rate of mutation.

Mitochondrial dysfunction can be part of the post-acute sequelae of COVID-19 [63]. Several studies have indicated that patients with post-COVID complications, showing chronic fatigue, neuropsychiatric and neurometabolic disturbances, could be affected by disorders of mitochondrial metabolic pathways [64-66]. An earlier study has shown that the mitochondrial membrane potential, a parameter used to evaluate the mitochondrial function, is decreased in leukocytes of subjects both after one and eleven months from COVID-19 recovery compared to healthy controls [67].

This study has some limitations that should be mentioned. First, the population analyzed is not vaccinated and a comparison with vaccinated individuals is missing. At this point of pandemic evolution there is a considerable part of the world population that has received at least one dose of a COVID-19 vaccine; therefore, it would be interesting to evaluate the consequences of the disease on mtDNA sequence also in vaccinated patients and analyze the possible differences with unvaccinated ones. Second, the validation from technical replicates or the use of another approach to verify findings from MitoChip analysis is lacking. Therefore, further confirmation using a more sensitive sequencing method would greatly enhance the reach of the present results especially those concerning heteroplasmy. Third, the mean age difference between COVID-19 patients and controls, with patients being significantly older than controls, could be a serious problem in interpreting the significance of the statistical analysis due to the increase of mtDNA heteroplasmic variants with age. To address this issue, we applied a PSM approach to all the statistical tests involving the comparison of the two groups, so as to control the age difference influence on test outcome. Finally, the small sample size limits the generalizability of our results that need to be confirmed in a larger cohort.

In conclusion, our findings suggest that mitochondria, particularly mtDNA, could be crucial targets of Sars-CoV-2 and might play an important role in the extent of viral invasiveness and pathogenicity. Although our study is cross sectional and direct causality between Sars-CoV-2 invasion and increase of damaging variants cannot be established, it can be argued that the effects of an inefficient oxidative phosphorylation in COVID-19 elderly patients could critically worsen an already compromised clinical status, with unpredictable consequences. Damage to mitochondria in COVID-19 could be long-lasting and affect general health conditions after recovery.

## CONCLUSIONS

Among the devastating effects of COVID-19, damage to mitochondria and mtDNA could have wide-ranging consequences and strongly weaken several metabolic pathways. Based on the emerging evidence, it is tempting to speculate that mitochondrial dysfunction may be the primary event in COVID-19 pathogenesis. In this perspective, elderly population, already suffering from mitochondrial dysfunction, represent the most exposed group. This suggests that COVID-19 may be targeted, in addition to antiviral strategies, by approaches that target oxidative phosphorylation, mitochondrial-mediated apoptosis, mitochondrial dynamics as well as the aging process.

## Supplementary Materials

The Supplementary data can be found online at: www.aginganddisease.org/EN/10.14336/AD.2023.1123.
