# Supplementary material for: Association of Inflammatory Mediators with Mitochondrial DNA Variants in Geriatric COVID-19 Patients
Source: Aging Dis. 2024 Feb 9;15(6):2665–81. doi: 10.14336/AD.2023.1123 (PMC11567249; doi:10.14336/AD.2023.1123)
Supplement: Supplementary file 1 — The Supplementary data can be found online at: www.aginganddisease.org/EN/10.14336/AD.2023.1123. [file AD-15-6-2665-s.pdf]

SUPPLEMENTARY DATA

Association of Inflammatory Mediators with Mitochondrial DNA Variants in Geriatric COVID-19 Patients

Tiziana Casoli, Anna Rita Bonfigli, Mirko Di Rosa, Belinda Giorgetti, Marta Balietti, Robertina Giacconi, Maurizio Cardelli, Francesco Piacenza, Francesca Marchegiani, Fiorella Marcheselli, Rina Recchioni, Roberta Galeazzi, Salvatore Vaiasicca, Adrianapia Maria Lamedica, Alessia Fumagalli, Letizia Ferrara, Fabrizia Lattanzio

© 2023. Casoli T et al. Published online at http://www.aginganddisease.org/EN/10.14336/AD.2023.1123

SUPPLEMENTARY DATA

Supplementary Table 1. Number of homoplasmic and heteroplasmic variants per individual**.**

Homoplasmic variants COVID-19

	339	352	384	415	416	419	429	437	448	449	453	464	475	484	495
**D-loop**	5	4	5	3	4	5	5	1	4	2	2	5	7	4	9
**12S**	1	2	2	3	2	3	3	2	1	2	2	3	4	3	2
**16S**	1		3	2		3	3			2	1	2	2		2
**ND1**			1	2		1	1	1	1	2		1	1		2
**ND2**	1	2	3	1	1	4	3	1		3	1	2	3		2
**COI**		2	1	2		2	1	1		2		1	1	1	2
**COII**															1
**ATPase8**	1														
**ATPase6**	2	1	2	2	1	3	2	1	1	1	1	2	3	1	2
**COIII**	1	1	1	1		3	1			1					1
**ND3**	1			1											2
**ND4L**				1		2									
**ND4**	3		2	5		3	4		1	2		4	5		2
**ND5**		1	3	3	3	2	2			4		2	1		3
**ND6**	2			1		1	1			1		1	2		
**Cytb**	2	2	2	3	1	3	5	3	2	2	1	4	4	1	3

	503	505	506	507	511	513	514	517	531	552	592	605	607	647	658
**D-loop**	7	8	9	5	7	2	10	7	10	8	7	5	2	4	2
**12S**	2	3	3	2	2	2	4	2	3	4	2	2	2	3	2
**16S**	3	2	2	2	2	1	2	2	2	2	2	1			1
**ND1**	2	1	1				1	1	1	1	1		1	1	1
**ND2**	5	2	3	2	1	2	2	2	2	2	1	2	1	1	2
**COI**	3	1	1	2	1		1	3	1	1	2	3		1	
**COII**					1			1		1					
**ATPase8**														1	
**ATPase6**	1	3	2	1	1	1	2	1	2	2	1	1	1	1	1
**COIII**			1		1		1		1	1		1			
**ND3**								1			1				
**ND4L**															
**ND4**	3	2	3	2	3		2	2	2	1	2	3			
**ND5**	2	3	2	2	2		1	1	2	2	2		1		
**ND6**					1						1				1
**Cytb**	2	6	5	3	2	1	5	3	5	5	3	1	1	2	1

Homoplasmic variants Controls

	1C	2C	3C	4C	5C	6C	7C	8C	9C	10C	11C	12C	13C	14C	15C
**D-loop**	9	3	9	2	8	2	3	2	7	3	3	7	6	5	6
**12S**	4	2	2	1	2	2	2	2	2	2	2	2	2	3	2
**16S**	2	1	1		1	1			3		1	3	2	2	3
**ND1**	1			1					1				1	1	2
**ND2**	3	2	1		2	2	1	1	1	1	2	3	1	2	2
**COI**	3	1	2		2		1	1	1		1	2	1	2	1
**COII**												1		1	
**ATPase8**															
**ATPase6**	2	2	1	1	2	1	2	2	1	1	1	2	1	1	1
**COIII**														2	
**ND3**			2						1				1		
**ND4L**															
**ND4**	3		3	1	3				2			3	2	3	2
**ND5**	3		2		1	1			2			2	2	1	2
**ND6**					1									1	
**Cytb**	3	1	4	2	3	1	1	1	3	1	1	3	3	4	4

© 2023. Casoli T et al. Published online at http://www.aginganddisease.org/EN/10.14336/AD.2023.1123

SUPPLEMENTARY DATA

	16C	17C	18C	19C	20C	21C	22C	23C	24C	25C	26C	27C	28C	29C	30C
**D-loop**	3	3	3	7	5	4	7	3	6	9	4	4	1	7	5
**12S**	2	2	2	2	2	2	3	2	2	2	2	2	3	2	1
**16S**			1	2	1		2	1	3	1	3	1	2	2	
**ND1**	1			1			1			3	1	1	1		1
**ND2**	1	2	1	2	1	1	1	5	2	2	2	2	2	2	
**COI**		1	1		1	1			1	1	1			2	1
**COII**			1							1					
**ATPase8**						1									
**ATPase6**	1	1	1	1	1	1	2	1	1	1	2	2	2	1	1
**COIII**				1		1	1				2	1			
**ND3**							1								
**ND4L**							1				2				
**ND4**				1			5	1	2		3	2	2	2	
**ND5**		2				1	2	1	4		2	1	4	4	1
**ND6**				1			1		1	2	1			1	
**Cytb**	1	2	1	2	1	1	3	2	2	1	3	1	5	3	2

	31C	32C	33C
**D-loop**	5	7	6
**12S**	4	2	2
**16S**	2		2
**ND1**	1		1
**ND2**	3	1	3
**COI**		2	
**COII**			
**ATPase8**			
**ATPase6**	1	1	2
**COIII**	1	1	1
**ND3**			
**ND4L**			
**ND4**	4		2
**ND5**	1		3
**ND6**	1		
**Cytb**	5	1	3

Heteroplasmic variants COVID-19

© 2023. Casoli T et al. Published online at http://www.aginganddisease.org/EN/10.14336/AD.2023.1123

SUPPLEMENTARY DATA

	339	352	384	415	416	419	429	437	448	449	453	464	475	484	495
**D-loop**	12	1	1		1	4	1	2	2	1		3			3
**12S**	4		1		1	1	1					1	1		
**16S**	3		3			1									
**ND1**	6		1	1		4			1	1		1			
**ND2**	7		1		2	3	2	1		2			1		
**COI**	10	1	1	1	1	11	1	2							
**COII**					1	2	1	4						1	
**ATPase8**															
**ATPase6**	4							1					1		
**COIII**	6	1	3	3	3	8	1	3	3	3		3	3	2	3
**ND3**	2					3									2
**ND4L**	2					3		2							
**ND4**	2		1	1		4	3	1	1	1			3		1
**ND5**	9	2			1	7	2	1							
**ND6**	5		1			1	2			2		1	2		
**Cytb**	3			1		1	5	1	1						1

	503	505	506	507	511	513	514	517	531	552	592	605	607	647	658
**D-loop**	3	4	2	1			2	4	2	2	1	1		4	
**12S**		1						2	1					1	
**16S**				1				2				1			1
**ND1**	2							2						2	1
**ND2**	1	1	1			2		4							
**COI**		1		1				4			1				
**COII**					1	1		2			1			1	
**ATPase8**														1	
**ATPase6**		2						1			1				
**COIII**	2	3	3	1	2	2	4	2	2	2	2	1	2	2	3
**ND3**								2			1	1			
**ND4L**											1				
**ND4**	1	1	1	1	2			3		1	1	1			
**ND5**					1	1		4			2			1	
**ND6**					1										
**Cytb**		1						2			1				

Heteroplasmic variants Controls

	1C	2C	3C	4C	5C	6C	7C	8C	9C	10C	11C	12C	13C	14C	15C
**D-loop**	2	2	5		3	1			4	1	1	4	1	1	3
**12S**	1		1											1	
**16S**					1	1						1			
**ND1**									1						1
**ND2**	1		1		2	2									
**COI**			1									1	1		
**COII**						1								1	
**ATPase8**															
**ATPase6**						1									
**COIII**	1	1	1	1	1		1	1	1	1	1	1	1		1
**ND3**															
**ND4L**															
**ND4**	1		1		2							1		1	2
**ND5**	1													3	2
**ND6**					1										
**Cytb**			2	1					1						1

	16C	17C	18C	19C	20C	21C	22C	23C	24C	25C	26C	27C	28C	29C	30C

© 2023. Casoli T et al. Published online at http://www.aginganddisease.org/EN/10.14336/AD.2023.1123

SUPPLEMENTARY DATA

**D-loop**	3		1	7	5	4	7	3	6	9	4	4	1	7	5
**12S**					1						1	1			
**16S**							1	1		3	2			1	
**ND1**										3		3			1
**ND2**					2			2		3	1	1			
**COI**							1	1			2	1		1	1
**COII**										1					2
**ATPase8**						1									
**ATPase6**										1	1				
**COIII**	1		1	1	1	1	2	2	3	2	5	4	1	2	1
**ND3**															
**ND4L**										1					
**ND4**	3						1			2	2		1	1	1
**ND5**		1						1		1	1	2	1		4
**ND6**					1		1								
**Cytb**					1	1	1								3

	31C	32C	33C
**D-loop**	5	7	6
**12S**	2		
**16S**			1
**ND1**			
**ND2**			1
**COI**			
**COII**			1
**ATPase8**			
**ATPase6**			
**COIII**	2	2	1
**ND3**			
**ND4L**			
**ND4**			1
**ND5**	1		2
**ND6**	1		
**Cytb**	1		

© 2023. Casoli T et al. Published online at http://www.aginganddisease.org/EN/10.14336/AD.2023.1123

SUPPLEMENTARY DATA

Supplementary Table 2. List of np which resulted significantly different in the comparison of the REA values between controls and COVID-19 patients. The position in the Mitochip v.2 (Probe set), GenBank, and corresponding genes are indicated, as well as the reference base, REA mean values, and age-weighted *p* values.

Probe Set	GenBank	Gene	Ref.	Control REA Mean (n=33)	COVID-19 REA Mean (n=30)	Age-weighted *p*-value
**human_mtDNA_RCRS-14103**	14105	ND5	c	0.68117	0.28584	3.73012E-10
**human_mtDNA_RCRS-14101**	14103	ND5	c	0.72382	0.33493	4.75351E-08
**human_mtDNA_RCRS-10457**	10459	tRNA arginine	a	0.64105	0.25417	8.0627E-06
**human_mtDNA_RCRS-2142**	2143	16S	g	0.97764	0.60216	3.60632E-12
**human_mtDNA_RCRS-15453**	15455	Cytb	c	0.68564	0.32483	4.44418E-07
**human_mtDNA_RCRS-1444**	1445	12S	g	0.94755	0.58717	3.27718E-07
**human_mtDNA_RCRS-14105**	14107	ND5	t	0.66454	0.30519	4.64461E-12
**human_mtDNA_RCRS-2110**	2111	16S	c	0.86238	0.50602	3.66971E-05
**human_mtDNA_RCRS-15451**	15453	Cytb	t	0.62558	0.27345	2.24187E-06
**human_mtDNA_RCRS-11111**	11113	ND4	t	0.92050	0.56979	2.86389E-08
**human_mtDNA_RCRS-10456**	10458	tRNA arginine	c	0.69321	0.34328	0.000135811
**human_mtDNA_RCRS-14107**	14109	ND5	c	0.70807	0.35815	3.13763E-10
**human_mtDNA_RCRS-1441**	1442	12S	g	0.84128	0.49326	1.64063E-08
**human_mtDNA_RCRS-14104**	14106	ND5	t	0.59498	0.24799	8.51934E-13
**human_mtDNA_RCRS-10454**	10456	tRNA arginine	a	0.51774	0.17120	1.63056E-05
**human_mtDNA_RCRS-5491**	5493	ND2	t	0.96486	0.61886	8.21792E-06
**human_mtDNA_RCRS-2148**	2149	16S	g	1.13194	0.78613	6.10614E-11
**human_mtDNA_RCRS-1443**	1444	12S	a	0.54098	0.19697	6.09526E-10
**human_mtDNA_RCRS-14097**	14099	ND5	t	0.82222	0.48056	1.44476E-08
**human_mtDNA_RCRS-15459**	15461	Cytb	t	0.80147	0.46711	7.72504E-07
**human_mtDNA_RCRS-1442**	1443	12S	t	0.57330	0.24051	1.74098E-09
**human_mtDNA_RCRS-15449**	15451	Cytb	c	0.56356	0.23121	6.21723E-08
**human_mtDNA_RCRS-1435**	1436	12S	c	0.52062	0.18830	1.63376E-06
**human_mtDNA_RCRS-8330**	8332	tRNA lysine	a	0.85322	0.52217	3.07989E-05
**human_mtDNA_RCRS-2109**	2110	16S	a	0.79766	0.46696	1.39555E-05
**human_mtDNA_RCRS-14106**	14108	ND5	t	0.57319	0.24332	3.56187E-13
**human_mtDNA_RCRS-7529**	7531	tRNA aspartic acid	a	0.94860	0.62223	9.17374E-05
**human_mtDNA_RCRS-15448**	15450	Cytb	t	0.58945	0.26538	6.86061E-08
**human_mtDNA_RCRS-1445**	1446	12S	a	0.95871	0.63645	1.37214E-06
**human_mtDNA_RCRS-1446**	1447	12S	g	1.14627	0.82499	7.48604E-07
**human_mtDNA_RCRS-16015**	16017	tRNA proline	t	0.90059	0.57973	3.69956E-08
**human_mtDNA_RCRS-11380**	11382	ND4	c	0.83174	0.51137	0.000108117
**human_mtDNA_RCRS-5515**	5517	tRNA tryptophan	t	0.73943	0.42092	4.00785E-05
**human_mtDNA_RCRS-9996**	9998	tRNA glycine	t	0.81644	0.49980	5.52919E-05
**human_mtDNA_RCRS-14095**	14097	ND5	c	1.00994	0.69331	1.35386E-05
**human_mtDNA_RCRS-5492**	5494	ND2	t	0.86553	0.55007	8.95026E-06
**human_mtDNA_RCRS-5513**	5515	ND2	a	0.93973	0.62771	0.000106993
**human_mtDNA_RCRS-4776**	4778	ND2	a	1.10826	0.79664	2.82703E-05
**human_mtDNA_RCRS-4775**	4777	ND2	c	0.97044	0.66007	5.28305E-05
**human_mtDNA_RCRS-10453**	10455	tRNA arginine	a	0.47121	0.16201	3.77605E-05
**human_mtDNA_RCRS-11110**	11112	ND4	t	0.79629	0.48942	5.92719E-07
**human_mtDNA_RCRS-5514**	5516	tRNA tryptophan	a	0.88585	0.58163	7.97506E-05
**human_mtDNA_RCRS-1434**	1435	12S	a	0.54036	0.23772	1.72954E-06
**human_mtDNA_RCRS-14108**	14110	ND5	t	0.70726	0.40488	8.58931E-09
**human_mtDNA_RCRS-4212**	4214	ND1	g	1.00025	0.69807	0.000541805
**human_mtDNA_RCRS-8741**	8743	ATPase6	g	0.87103	0.57079	2.18943E-05
**human_mtDNA_RCRS-14098**	14100	ND5	c	0.77648	0.47681	1.66482E-06
**human_mtDNA_RCRS-2296**	2297	16S	a	1.00871	0.70978	0.000112314
**human_mtDNA_RCRS-12138**	12140	tRNA histidine	a	0.96296	0.66461	2.24259E-06
**human_mtDNA_RCRS-5516**	5518	tRNA tryptophan	t	0.95758	0.65977	0.000142556
**human_mtDNA_RCRS-2299**	2300	16S	g	1.20655	0.90908	0.000129414
**human_mtDNA_RCRS-9998**	10000	tRNA glycine	g	0.77613	0.47917	0.000613666
**human_mtDNA_RCRS-5499**	5501	ND2	a	0.61906	0.32312	7.98154E-05
**human_mtDNA_RCRS-15452**	15454	Cytb	t	0.56812	0.27243	2.48427E-05
**human_mtDNA_RCRS-14099**	14100	ND5	c	0.79844	0.50364	8.36083E-06
**human_mtDNA_RCRS-16012**	16014	D-loop	c	1.09843	0.80632	1.86758E-05
**human_mtDNA_RCRS-14109**	14111	ND5	t	0.72920	0.43833	8.65213E-08
**human_mtDNA_RCRS-7527**	7529	tRNA aspartic acid	a	0.89955	0.60951	0.000195722
**human_mtDNA_RCRS-2307**	2309	16S	a	0.71968	0.43108	0.000136718
**human_mtDNA_RCRS-7567**	7569	tRNA aspartic acid	a	0.70991	0.42187	0.000537533
**human_mtDNA_RCRS-7306**	7308	COI	a	0.77941	0.49260	0.000264886

© 2023. Casoli T et al. Published online at http://www.aginganddisease.org/EN/10.14336/AD.2023.1123

SUPPLEMENTARY DATA

**human_mtDNA_RCRS-2108**	2110	16S	a	0.90855	0.62264	0.000118478
**human_mtDNA_RCRS-15458**	15460	Cytb	c	0.77941	0.49355	9.90068E-07
**human_mtDNA_RCRS-15331**	15333	Cytb	a	0.65074	0.36514	2.4354E-08
**human_mtDNA_RCRS-10235**	10237	ND3	t	1.16580	0.88025	3.20631E-06
**human_mtDNA_RCRS-9997**	9999	tRNA glycine	a	0.48930	0.20379	7.08146E-06
**human_mtDNA_RCRS-10450**	10452	tRNA arginine	g	0.54479	0.25942	0.000196543
**human_mtDNA_RCRS-8494**	8496	ATPase8	t	0.85591	0.57224	1.06746E-05
**human_mtDNA_RCRS-7569**	7571	tRNA aspartic acid	a	0.87793	0.59461	0.000443221
**human_mtDNA_RCRS-15330**	15332	Cytb	c	0.69095	0.40844	3.10417E-07
**human_mtDNA_RCRS-9995**	9997	tRNA glycine	t	0.95569	0.67368	0.000116079
**human_mtDNA_RCRS-2308**	2310	16S	a	0.78581	0.50405	0.000155348
**human_mtDNA_RCRS-10234**	10236	ND3	a	0.98009	0.69974	1.60993E-06
**human_mtDNA_RCRS-10458**	10460	tRNA arginine	t	0.71402	0.43428	0.000178425
**human_mtDNA_RCRS-2294**	2295	16S	c	1.04448	0.77061	0.000342245
**human_mtDNA_RCRS-5088**	5090	ND2	t	0.66298	0.38976	0.000136946
**human_mtDNA_RCRS-1440**	1441	12S	a	0.72136	0.44908	2.89053E-07
**human_mtDNA_RCRS-2295**	2296	16S	t	0.88872	0.61644	0.000364765
**human_mtDNA_RCRS-14775**	14777	Cytb	a	0.84760	0.57637	9.82412E-05
**human_mtDNA_RCRS-7528**	7530	tRNA aspartic acid	a	0.90658	0.63539	0.000548359
**human_mtDNA_RCRS-2144**	2145	16S	g	0.94360	0.67241	6.47458E-09
**human_mtDNA_RCRS-4213**	4215	ND1	a	0.83967	0.56942	0.000794312
**human_mtDNA_RCRS-5078**	5080	ND2	a	0.96566	0.69696	7.11885E-05
**human_mtDNA_RCRS-15455**	15457	Cytb	c	0.68710	0.41873	4.77058E-06
**human_mtDNA_RCRS-10231**	10233	ND3	g	0.98032	0.71312	2.87575E-05
**human_mtDNA_RCRS-5512**	5514	tRNA tryptophan	a	0.88755	0.62147	0.000282906
**human_mtDNA_RCRS-4756**	4758	ND2	a	0.87073	0.60476	0.000276658
**human_mtDNA_RCRS-6362**	6364	COI	g	0.65008	0.38518	3.92963E-05
**human_mtDNA_RCRS-10238**	10240	ND3	c	1.18575	0.92098	7.13502E-05
**human_mtDNA_RCRS-5093**	5095	ND2	t	0.87895	0.61469	5.81499E-05
**human_mtDNA_RCRS-7517**	7519	tRNA aspartic acid	a	0.71535	0.45170	0.000524756
**human_mtDNA_RCRS-10217**	10219	ND3	a	1.17188	0.90842	4.53913E-07
**human_mtDNA_RCRS-4774**	4776	ND2	g	0.64809	0.38464	0.000455623
**human_mtDNA_RCRS-1433**	1434	12S	a	0.55668	0.29346	9.26815E-05
**human_mtDNA_RCRS-5092**	5094	ND2	a	0.66520	0.40287	1.39763E-05
**human_mtDNA_RCRS-6227**	6229	COI	t	0.43395	0.17217	7.09846E-08
**human_mtDNA_RCRS-4290**	4291	tRNA isoleucine	g	1.21505	0.95370	3.33475E-05
**human_mtDNA_RCRS-6224**	6226	COI	c	0.49451	0.23320	3.32481E-09
**human_mtDNA_RCRS-6228**	6230	COI	c	0.49178	0.23085	5.63862E-09
**human_mtDNA_RCRS-237**	238	D-loop	a	0.98973	0.72918	0.000333001
**human_mtDNA_RCRS-5506**	5508	ND2	t	0.51040	0.24989	0.000171665
**human_mtDNA_RCRS-6226**	6228	COI	c	0.51670	0.25676	6.93672E-09
**human_mtDNA_RCRS-14096**	14098	ND5	t	0.83077	0.57124	2.2191E-05
**human_mtDNA_RCRS-5075**	5077	ND2	t	0.86846	0.60918	3.39309E-05
**human_mtDNA_RCRS-14094**	14096	ND5	a	0.74387	0.48532	1.31646E-05
**human_mtDNA_RCRS-10111**	10113	ND3	a	0.48499	0.22714	9.57269E-05
**human_mtDNA_RCRS-2297**	2298	16S	a	1.08742	0.82978	3.96069E-05
**human_mtDNA_RCRS-6361**	6363	COI	g	0.59802	0.34116	2.63637E-05
**human_mtDNA_RCRS-10455**	10457	tRNA arginine	t	0.52504	0.26825	5.9007E-05
**human_mtDNA_RCRS-4292**	4293	tRNA isoleucine	t	0.92783	0.67202	1.82727E-06
**human_mtDNA_RCRS-10487**	10489	ND4L	a	0.85489	0.59924	0.000379206
**human_mtDNA_RCRS-4217**	4218	ND1	g	0.98548	0.73000	0.00054503
**human_mtDNA_RCRS-8745**	8747	ATPase6	c	1.14948	0.89451	0.00044838
**human_mtDNA_RCRS-4777**	4779	ND2	a	1.18547	0.93066	0.000201927
**human_mtDNA_RCRS-11113**	11115	ND4	a	0.82781	0.57320	7.60966E-06
**human_mtDNA_RCRS-5497**	5499	ND2	c	0.79188	0.53732	0.000661548
**human_mtDNA_RCRS-14102**	14104	ND5	t	0.53583	0.28267	1.89363E-09
**human_mtDNA_RCRS-10007**	10009	tRNA glycine	g	1.24436	0.99237	0.000165092
**human_mtDNA_RCRS-9994**	9996	tRNA glycine	t	1.01878	0.76695	0.000100888
**human_mtDNA_RCRS-11115**	11117	ND4	a	0.89578	0.64407	2.01528E-05

© 2023. Casoli T et al. Published online at http://www.aginganddisease.org/EN/10.14336/AD.2023.1123
